# The lethal cargo of *Myxococcus xanthus* outer membrane vesicles

**DOI:** 10.3389/fmicb.2014.00474

**Published:** 2014-09-09

**Authors:** James E. Berleman, Simon Allen, Megan A. Danielewicz, Jonathan P. Remis, Amita Gorur, Jack Cunha, Masood Z. Hadi, David R. Zusman, Trent R. Northen, H. Ewa Witkowska, Manfred Auer

**Affiliations:** ^1^Life Sciences Division, Lawrence Berkeley National LaboratoryBerkeley, CA, USA; ^2^Department of Molecular and Cell Biology, University of California, BerkeleyBerkeley, CA, USA; ^3^School of Biology, St. Mary's CollegeMoraga, CA, USA; ^4^Department of Obstetrics, Gynecology and Reproductive Science, UCSF Sandler-Moore Mass Spectrometry Core FacilitySan Francisco, CA, USA; ^5^Space Biosciences Division, Synthetic Biology Program, NASA Ames Research CenterMoffett Field, CA, USA; ^6^Physical Biosciences Division, Lawrence Berkeley National LaboratoryBerkeley, CA, USA

**Keywords:** predation, fruiting body, predatory rippling, predator-prey interactions, secondary metabolism and enzymes

## Abstract

*Myxococcus xanthus* is a bacterial micro-predator known for hunting other microbes in a wolf pack-like manner. Outer membrane vesicles (OMVs) are produced in large quantities by *M. xanthus* and have a highly organized structure in the extracellular milieu, sometimes occurring in chains that link neighboring cells within a biofilm. OMVs may be a vehicle for mediating wolf pack activity by delivering hydrolytic enzymes and antibiotics aimed at killing prey microbes. Here, both the protein and small molecule cargo of the OMV and membrane fractions of *M. xanthus* were characterized and compared. Our analysis indicates a number of proteins that are OMV-specific or OMV-enriched, including several with putative hydrolytic function. Secondary metabolite profiling of OMVs identifies 16 molecules, many associated with antibiotic activities. Several hydrolytic enzyme homologs were identified, including the protein encoded by MXAN_3564 (*mepA*), an M36 protease homolog. Genetic disruption of *mepA* leads to a significant reduction in extracellular protease activity suggesting MepA is part of the long-predicted (yet to date undetermined) extracellular protease suite of *M. xanthus*.

## Introduction

The outer membrane (OM) of bacteria plays a crucial role in mediating cell-cell interactions for symbiotic and pathogenic relationships (Marshall, 2005; Cross, 2014). The OM provides a permeable barrier with important functions in transport and protection for the cell envelope of Gram-negative bacteria (Nikaido, 2003; Cornejo et al., 2014). Though the OM is often generalized, there is a great deal of diversity in this envelope structure that may help inform us on the evolutionary origin of the OM and the functional capacity of this structure (Vollmer, 2012; Jiang et al., 2014). One example of this OM diversity is the biosynthesis by some bacteria of OM vesicles (OMVs) (Beveridge, 1999; Mashburn-Warren et al., 2008), and more recently vesicle chains and tubes (Remis et al., 2014). OMVs provide a variety of additional functions to bacterial cells that go beyond the traditional concept of the OM, including the exchange of beneficial cell-cell communication signals, delivery of harmful toxins in pathogenesis and microbial competition (Kesty et al., 2004; Mashburn and Whiteley, 2005; Evans et al., 2012). The larger, more complex chain and tube structures can also provide a network function analogous to mycelia and filamentous multicellular organizations (Wei et al., 2011; Remis et al., 2014).

Here, we examine the OMV cargo of *Myxococcus xanthus*, a delta-proteobacterium that produces copious amounts of OM-derived structures. *M. xanthus* is a common soil bacterium that displays complex social behavior through the formation of multicellular structures that facilitate surface colonization during vegetative swarming, segregation of cell types through aggregation into fruiting bodies for differentiation into spores as well as predation of prey microorganisms (Berleman et al., 2006; Pelling et al., 2006; Velicer and Vos, 2009). The “wolf pack” predatory behavior of *M. xanthus* is of particular interest as cells are able to distinguish between self and non-self even when attacking other Gram-negative bacteria such as *E. coli* by lysing prey bacteria without the aid of phagocytosis (Berleman et al., 2008; Pan et al., 2013). The “wolf pack” response is observed during predataxis, where the judicious oscillation of cell reversals directs *M. xanthus* cell movement through prey colonies. The killing of *E. coli* cells has long been thought to depend on the secretion of antibiotics and proteolytic exoenzymes (Rosenberg et al., 1977). Here, we utilize liquid chromatography-mass spectrometry (LC-MS) approaches to define the molecular cargo that mediates the wolf pack predatory ability of *M. xanthus*.

The *M. xanthus* genome is large for bacteria, with 7331 predicted protein coding loci (Goldman et al., 2006). The large genome size is due in part to a large reservoir of genes coding for hydrolytic enzymes and secondary metabolite pathways, both of which are thought to benefit the predatory life style of this microbe. A wide variety of secondary metabolites have been identified from *M. xanthus* and related organisms including natural products with antibiotic, antifungal and anti-tumor activity (Gerth et al., 1983; Krug et al., 2008; Li et al., 2014). Myxovirescin (also called antibiotic TA), the myxochelins and the myxalamids produced by *M. xanthus* all have antibiotic properties, but their expression and native function are not well understood (Gerth et al., 1983; Li et al., 2008; Xiao et al., 2012). The extracellular proteins that localize to matrix polysaccharides have previously been profiled, with one locus identified that is required for fruiting body development in certain backgrounds (Curtis et al., 2007). The protein profile of OMVs has been shown to differ between high nutrient and low nutrient conditions (Kahnt et al., 2010), but there is still little known about extracellular enzymatic activity in *M. xanthus*. Here, we identify a protease in *M. xanthus* similar to M36 fungalysins utilized by fungi for extracellular hydrolytic activity (Lilly et al., 2008).

## Results

### Fractionation of vesicles from outer membranes of *M. xanthus*

**Figure 1:**
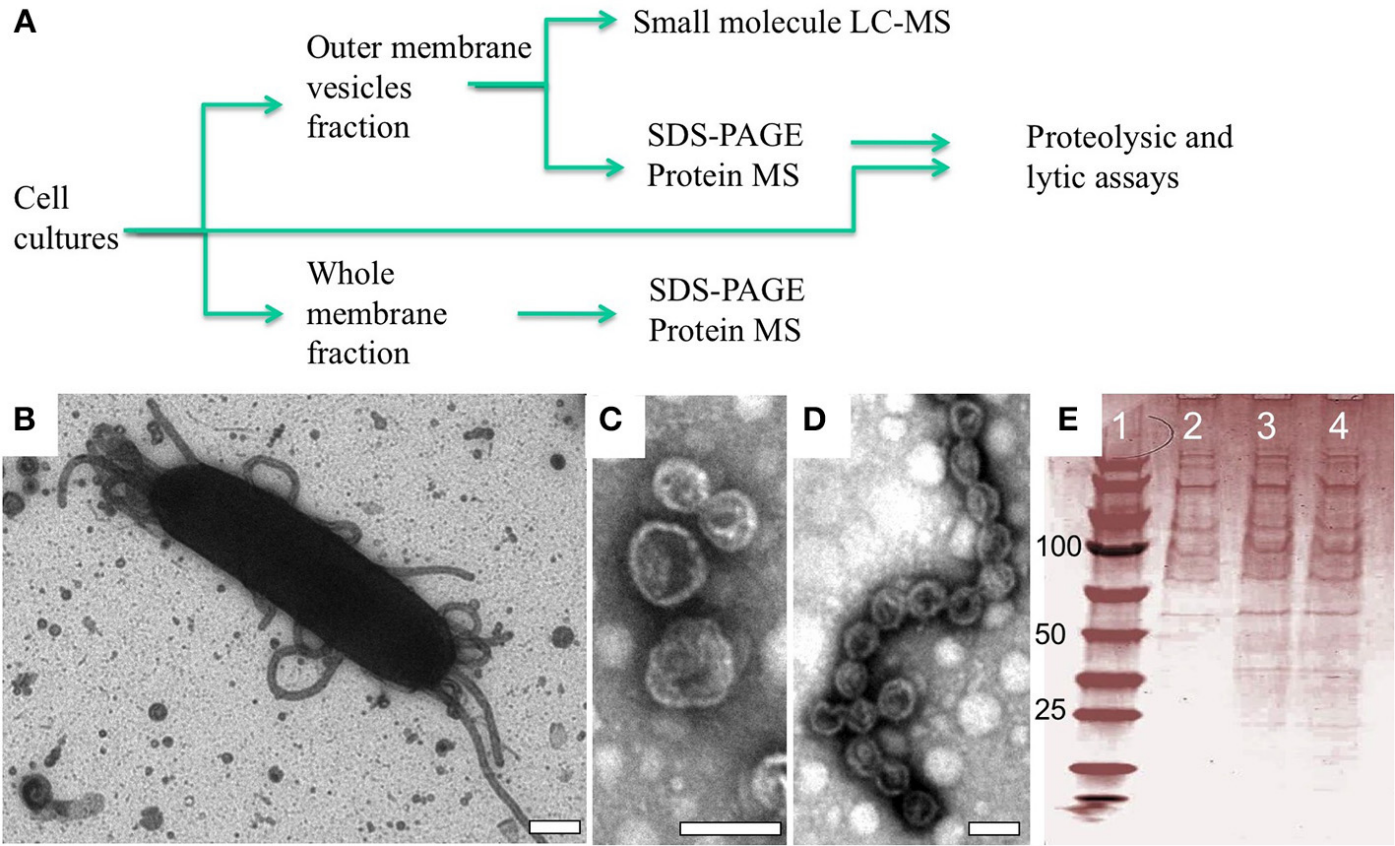
**OMV structures observed *in situ* and after purification. (A)** Flow chart of experimental procedure with representative analytics **(B–E)**. **(B)** Negative staining EM of an *M. xanthus* wild type DZ2 cell showing a mixture of extracellular structures associated with the cell including isolated vesicles and vesicle chains (Scale bar = 200 nm). After purification, these structures maintain their distinctive shape as **(C)** vesicles and **(D)** vesicle chains (Scale bars = 40 nm). **(E)** SDS-PAGE analysis showing consistency of protein profiles in OMV fractions used for protein MS: lane 1 standards, lane 2–4 OMV fractions from 3 independent cultures.

OMV structures have been observed in a number of bacteria, but the full extent of their function(s) remains a topic of study. Lab cultures of *M. xanthus* produce OMVs, some of which remain tethered to the cell surface, whereas others are secreted into the extracellular environment (Palsdottir et al., 2009). The chemical composition of OMVs has been shown to include lipid, carbohydrate and protein macromolecules (Kahnt et al., 2010; Evans et al., 2012; Remis et al., 2014), all of which likely play a critical role in OMV organization and possibly in fusion of OMVs to other cells. To determine the secondary metabolite and protein cargo of OMVs we isolated and purified OM and OMV fractions, respectively, from wild type cells using ultracentrifugation and serial filtration (Figure 1A). The presence of OMVs structures were confirmed by both transmission electron microscopy (TEM) of whole cells (Figure 1B) and after OMV purification (see Figures 1C,D) and normalized via quantifying incorporation of the lipophilic dye FM4-64 as described previously (Remis et al., 2014). This confirmed that the integrity of the vesicle structures was maintained throughout the preparation (see Figures 1C,D). SDS-PAGE analysis of our OMV preparations indicates a large number of proteins in this fraction, in agreement with previous studies on extracellular fractionation (Kahnt et al., 2010).

### Mass spectrometry of cell fractions identified a unique OMV protein cargo

**Figure 2:**
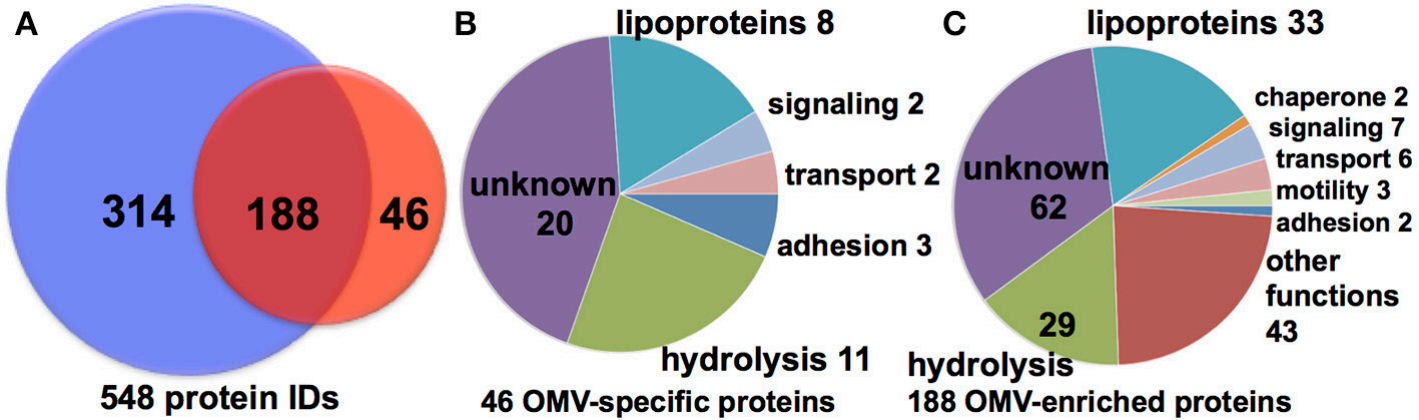
**Cell fraction comparison and predicted function. (A)** Conservative set of 548 (46 OMV only, 188 shared, 314 OM only) protein IDs from MS analysis of >10,000 peptides. Putative function binning of **(B)** 46 OMV-specific and **(C)** 188 OMV-enriched proteins.

To determine the protein cargo of each cell fraction, proteins isolated from purified vesicles and whole cell membranes were digested with trypsin and proteolytic peptides examined by reversed phase (RP) liquid chromatography (LC) mass spectrometry (MS). RP LCMS analysis of three OMV and membrane fractions from independent cultures delivered consistent results in terms of a number of matched peptides and identified proteins, summarized in Tables S1 and S2 (Supplement Data S1). The MS evidence for all protein identification in all samples based on peptides matched above the peptide identify threshold is shown in Supplement Data S2. A total of 287 and 786 proteins were identified in OMV and OM, respectively (Figure 2 and Table 1). Of these proteins, 88 proteins were detected only in the OMV fraction (S3), 199 were detected in both cell fractions (Table S4), and 583 proteins were detected only in the membrane fractions (S5). This comparison suggests that there is a protein cargo that is unique to OMVs. There are 7331 total predicted proteins in the *M. xanthus* complete genome (Goldman et al., 2006), indicating that we have detected 12 % of the total genomic potential in these two fractions. To limit further analysis to proteins that were identified with the highest stringency, we reduced this pool by considering only proteins that were identified in at least two samples on the basis of at least two distinct peptides This analysis yields a conservative set of 46 OMV-specific, 188 OMV-contained, and 314 membrane proteins, or 7.5 % of the total genome prediction (Figure 2A). (Nudleman et al., 2005; Ducret et al., 2013; Wei et al., 2014).

**Table 1:** **MS analysis of purified vesicles and purified membrane fraction**.

**Loci**	**Putative functions**
**OMV SPECIFIC**	
20 loci	Hypothetical proteins
8 loci	Putative lipoproteins
MXAN_0366	Endonuclease/exonuclease/phosphatase family protein
MXAN_0587	Trypsin domain protein
MXAN_0805	Peptidase, M10A/M12A subfamilies
MXAN_1650	Peptidase, S1A (chymotrypsin) subfamily
MXAN_1967	Putative peptidase, S8 (subtilisin) family
MXAN_2760	Peptidase, M28E family
MXAN_3162	Fumarate hydratase, class I
MXAN_3577	Metallophosphoesterase
MXAN_4534	Chitinase, class I
MXAN_5454	Peptidase, M36 (fungalysin) family
MXAN_5970	Peptidase, S8 (subtilisin) family
MXAN_0510	Putative cell wall surface anchor family protein
MXAN_4914	F5/8 type C domain protein
MXAN_5098	Putative Ig domain protein
MXAN_1668	Serine/threonine kinase associate protein
MXAN_5229	Putative GTP-binding protein
MXAN_0362	Putative pilus biogenesis operon protein
MXAN_0889	RCC1 repeat domain protein
**OMV ENRICHED**	
62 loci	Unknown
38 loci	Putative lipoproteins
43 loci	Other functions
MXAN_0201	Hydrolase, alpha/beta fold family
MXAN_0533	NAD dependent epimerase/dehydratase family
MXAN_0886	Metal dependent amidohydrolase
MXAN_1389	Putative alkaline phosphatase
MXAN_1394	Metallo-beta-lactamase family protein
MXAN_1564	Alkyl hydroperoxide reductase C
MXAN_1623	Peptidase, M16 (pitrilysin) family
MXAN_1624	Peptidase, M16 (pitrilysin) family
MXAN_2016	Prolyl endopeptidase precursor
MXAN_2382	Peptidase, M18 (aminopeptidase I) family
MXAN_2519	Peptidase U62
MXAN_2787	Homogentisate 1,2-dioxygenase
MXAN_2814	Putative N-acetylmuramoyl-L-alanine amidase
MXAN_2906	Penicillin acylase family protein
MXAN_3042	Glycine dehydrogenase
MXAN_3160	Peptidase, M13 (neprilysin) family
MXAN_3465	Histidine ammonia-lyase
MXAN_3564	Peptidase, M36 (fungalysin) family
MXAN_4146	Alanine dehydrogenase
MXAN_4327	Glu/Leu/Phe/Val dehydrogenase
MXAN_5040	Aldehyde dehydrogenase
MXAN_5326	Putative phytase
MXAN_5933	Peptidase, M48 (Ste24 endopeptidase) family
MXAN_6106	Matrix-associated zinc metalloprotease FibA
MXAN_6266	Putative 2,3-cyclic-nucleotide 2-phosphodiesterase
MXAN_6516	Adenosylhomocysteinase
MXAN_6539	Extracellular protease domain protein
MXAN_6601	Peptidase, S9C (acylaminoacyl-peptidase) subfamily
MXAN_4796	Fibronectin type III domain protein
MXAN_4467	Chaperonin GroEL1
MXAN_4895	Chaperonin GroEL2
MXAN_2538	Adventurous gliding motility protein AgmO
MXAN_3060	Adventurous gliding motility protein CglB
MXAN_4865	Adventurous gliding motility protein AgmV
MXAN_1450	TonB-dependent receptor
MXAN_4559	TonB dependent receptor
MXAN_5766	TPR domain protein
MXAN_6248	Cyclic nucleotide-binding domain protein
MXAN_6911	TonB-dependent receptor
MXAN_5042	OmpA domain protein
MXAN_5598	OmpA domain protein
MXAN_6665	Putative branched chain amino acid ABC transporter

Vesicle protein cargo can be broken down into two groups, proteins that are consistently detected in the OMV purified samples, but not the membrane samples (OMV specific) and proteins are consistently detected and shared between the two cell fractions (OMV enriched). Data was collected from 3 independent vesicle purifications and membrane purifications. Pertinent data is shown here, full data set is available in Supplement Data S1.

A majority of the identified OMV-specific proteins (20 of 46) are listed as uncharacterized hypothetical proteins, making functional prediction difficult (Figure 2B, Table 1). Of the remaining proteins identified in the purified OMV fraction, 8 are listed as putative lipoproteins and 11 have a putative hydrolytic activity. The latter include 7 proteins predicted to have peptidase or protease activity (MXAN_0587, MXAN_0805, MXAN_1650, MXAN_1967, MXAN_2760, MXAN_5454, and MXAN_5970). Chitinase (MXAN_4534), phosphoesterase (MXAN_3577), hydratase (MXAN_3162), and nuclease (MXAN_0366) enzymatic functions are also predicted. We also found several proteins predicted to be involved in extracellular functions, such as MXAN_0362 (putative pilus protein), MXAN_0889 (RCC1 repeat beta-propeller fold), and adhesins (MXAN_0510, and MXAN_4914). A similar distribution of functions was observed if we expand our analysis to the 234 OMV-enriched proteins (46 specific, 188 shared with membrane) detected (Figure 2C), with more hydrolytic functions, particularly peptidase and protease homologs. We also detected A-motility proteins, CglB, AgmO, and AgmV, supporting a role for OMVs in intra-species sharing of motility components, although it is still subject to debate whether OMVs are directly involved in the transfer of motility components between *M. xanthus* cells (Nudleman et al., 2005; Ducret et al., 2013; Wei et al., 2014). The detection of Agm motility proteins in membrane fractions supports previous findings (Luciano et al., 2011).

**Figure 3:**
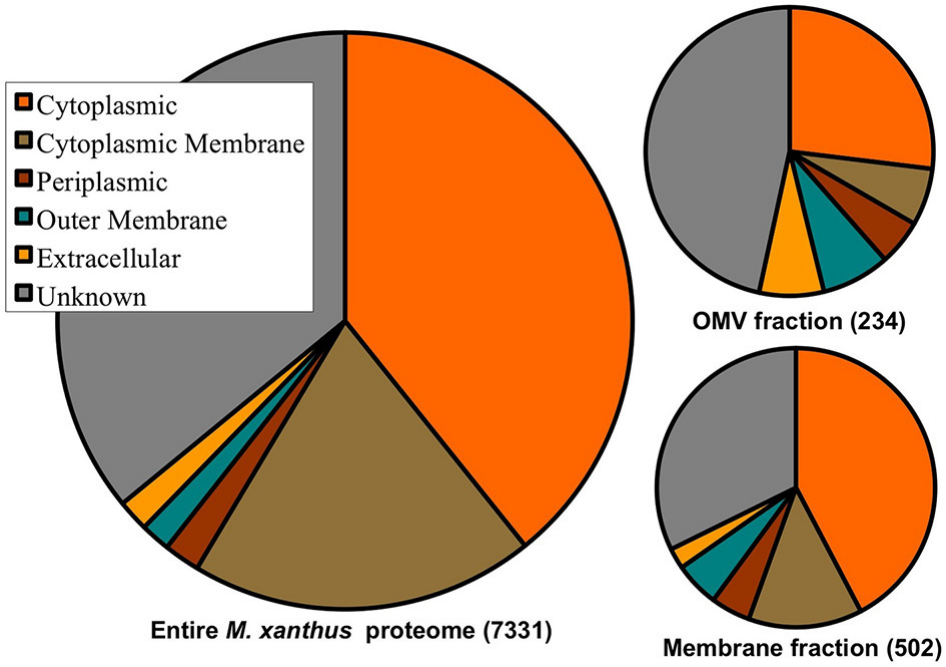
**Cell fraction vs. protein localization prediction comparison**. Distribution of cellular localization predictions of the entire 7331 *M. xanthus* proteome, the 234 proteins detected in the OMV preparations, and the 502 proteins detected in the membrane preparations, with 188 shared between the two. Protein localization predictions show enrichment for unknown, extracellular and OM proteins in OMVs.

Of the 7331 predicted proteins in the total *M. xanthus* proteome, cellular localization programs (e.g., PSORTb), give expected localization for ~66% of the proteome, with most proteins predicted to reside in the cytoplasm or cytoplasmic membrane (see Figure 3). By comparison, the proteins detected in the OMV fraction are enriched for proteins with “unknown localization.” While the membrane preparations are also enriched for OM and IM proteins, it is important to note that proteins we have identified in the OMV fraction come from all cell fractions based on the current predictions. This may be due to several factors: ubiquitous localization (e.g., proteins may localize to more than one fraction), misannotation (imperfect prediction algorithms), or contamination of a few proteins from another cell fraction during the purification process. However, the most abundant cytoplasmic proteins that are common artifacts of other cell fractions, e.g., ribosomal proteins (Choi et al., 2011), were rarely detected in our samples, indicating that cross contamination is least likely, and that this data will help to improve prediction programs.

### Abundance of OMV proteins

The relative abundance of each protein in the samples was approximated by calculating its contribution to a sum of the exponentially modified protein indices (emPAI) assigned to each sample component (mol %) (Ishihama et al., 2005). Thus, generated (mol %) value estimates of relative protein abundances in each sample were then compared across the samples (Table 2, Tables S2–S4). Out of six proteins found in OMV at relative abundance above 2%, four appeared at a higher relative level in OMV than in the corresponding OM samples, i.e., lipoproteins (MXAN_6367 and MXAN_7333), a hydrolase (MXAN_0201) and a hypothetical protein (MXAN_5453). The other two, chaperonins GroEL1 (MXAN__4895) and GroEL2 (MXAN__4467) ranked as the first and third most abundant protein in OMV, albeit were present at ~50% level of the observed in OM. Nineteen proteins were enriched by more than 10-fold in OMV compared with membrane (OM) samples. Of those, nine have unknown function (leading with MXAN_1365 at >75 fold excess), five are lipoproteins (leading with MXAN_2277 at >30 fold excess), and three are hydrolases (an alpha/beta fold hydrolase, MXAN_0201, an M36 peptidase, MXAN_3564, and metal dependent amidohydrolase, MXAN_0886, the latter at >65 fold excess). Of note, three TonB-dependent receptor related proteins were found in OMV fractions (MXAN_6911, MXAN_4559, and MXAN_1450). The presence of two GroEL chaperone homologs coded for in the *M. xanthus* genome have been investigated previously, and GroEL1 has been shown to be essential for fruiting body development and sporulation, whereas GroEL2 plays a role in antibiotic production and predation (Li et al., 2010; Wang et al., 2014). The abundance of GroEL chaperonins in the OMV fraction suggests the possibility that OMV proteins may require proper protein folding after export into OMV structures. Also, GroEL proteins are known to function in large tetradecamer complexes, which may in part explain the high frequency of detection. The presence of TonB-dependent receptor proteins and a large number of hydrolytic enzymes suggests that OMVs contain transport and catalytic functions.

**Table 2:** **Relative abundance of proteins detected in vesicles through semi-quantitative MS analysis**.

**Prot_acc**	**Putative function**	**OMV Avg Score**	**OMV mol %**	**OM Avg Score**	**OM mol %**	**Relative ratio OMV/OM**
MXAN_1365	Hypothetical protein	4448 ± 1517	1.3 ± 0.6	414 ± 62	0.02 ± 0.001	77.5
MXAN_4895	Chaperonin GroEL 2	4350 ± 908	8.2 ± 3.8	70526 ± 9769	16.3 ± 4.6	0.5
MXAN_1450	TonB-dependent receptor	3583 ± 875	1.6 ± 0.4	7244 ± 904	1 ± 0.2	1.6
MXAN_0962	Putative lipoprotein	3533 ± 628	1.2 ± 0.1	2760 ± 100	0.2 ± 0.01	5.8
MXAN_0201	Hydrolase, alpha/beta fold family	3272 ± 1094	2.6 ± 0.7	376 ± 313	0.2 ± 0.1	14.1
MXAN_5453	Hypothetical protein	2938 ± 979	2.6 ± 0.7	1368 ± 817	0.3 ± 0.2	9.4
MXAN_4467	Chaperonin GroEL 1	2515 ± 769	3.4 ± 1.1	33949 ± 6097	6.4 ± 1.6	0.5
MXAN_3564	Peptidase, M36 (fungalysin) family	2471 ± 737	0.4 ± 0.1	643 ± 430	0.04 ± 0.03	11.0
MXAN_4860	Hypothetical protein	2379 ± 383	0.8 ± 0.3	1162 ± 231	0.2 ± 0.01	3.7
MXAN_7199	Putative lipoprotein	1800 ± 711	0.4 ± 0.1	677 ± 95	0.1 ± 0.01	4.5

### Mass spectrometry analysis of small molecules in OMVs

**Figure 4:**
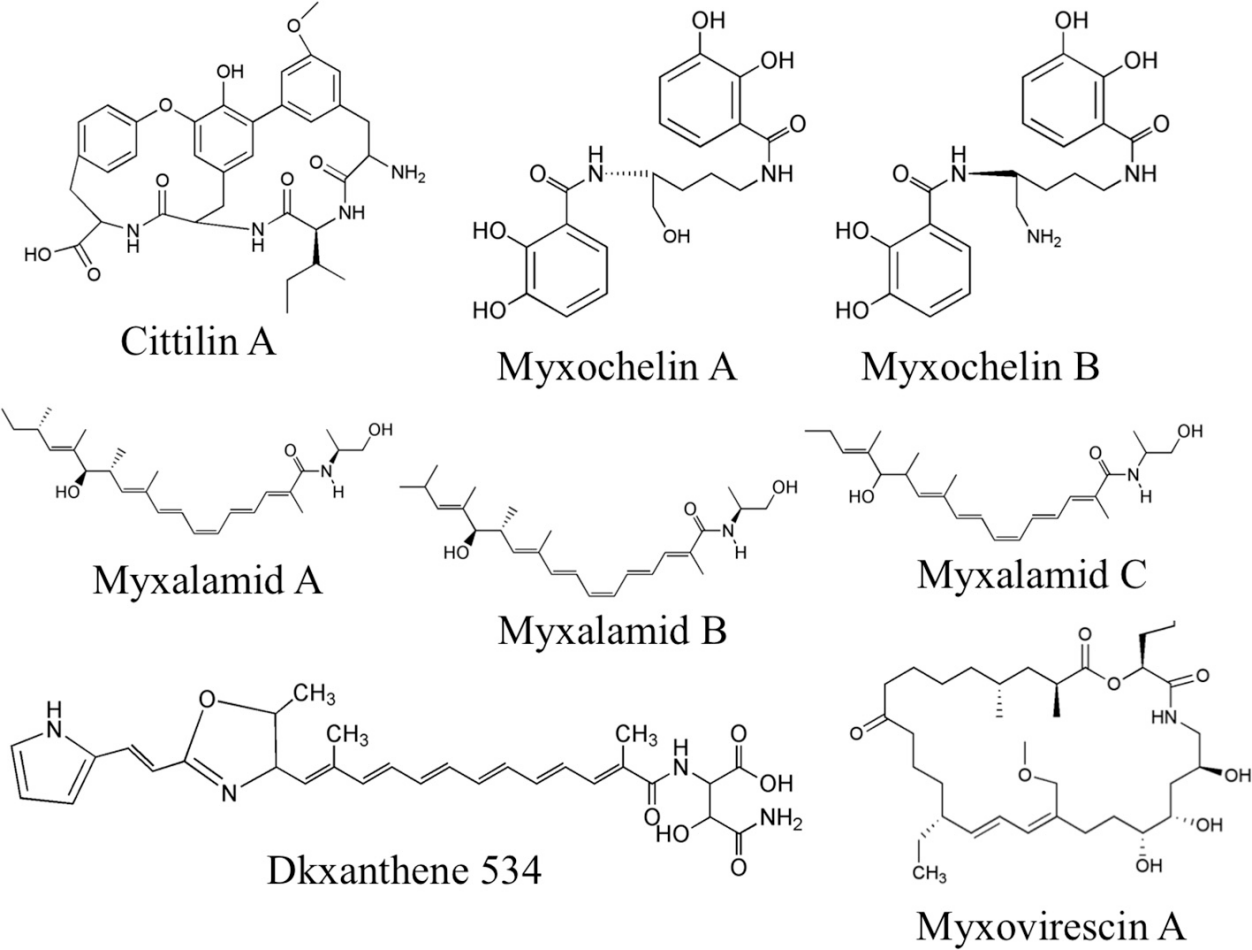
**Secondary metabolites detected in OMV fractions**. Representative structures from tandem MS analysis of *M. xanthus* OMV fractions are shown, based on comparison to known *M. xanthus* products. Six chemical families were identified, most of which have some antibiotic activity. Note that for the dkxanthene chemical family, nine distinct members were detected but only DKxanthene534 is shown. Further detail can be found in Table 3 and Supplement Table 2.

Purified OMV fractions of *M. xanthus* cultures were also prepared for analysis by Liquid Chromatography Mass spectrometry (LC/MS) for determination of secondary metabolites. Profiling was performed via targeted searches for known *M. xanthus* secondary metabolites (Krug et al., 2008; Kim et al., 2009; Weissman and Müller, 2010). Tandem MS (MS/MS) was performed to help identify 16 molecules from five chemical families based on accurate mass and MS/MS (Supplement Data S2). We detected and confirmed the presence in OMV fractions for two myxochelins (m/z of 404.183 and 405.167, respectively), three myxalamids (m/z of 416.317, 402.301, and 388.285, respectively), and nine dkxanthenes (m/z values ranging from 493.248 to 597.168) (Table 3 for MS details, Figure 4 for predicted structures). Myxochelins are iron chelating siderophores that have been shown, when purified, to have some antibacterial properties as well (Weissman and Müller, 2010). Myxochelin A can be synthesized from two molecules of 2,3-dihydroxybenzoic acid condensed with the amine groups of a lysine residue (Li et al., 2008). The myxalamids have desaturated linear or branched carbon backbones (C17–18 long), with hydroxyl residues at one end. The myxalamids have antibiotic properties by inhibiting the cytochrome I NADH:ubiquinone oxidoreductase (Gerth et al., 1983). Dkxanthenes have a common chemical backbone with many different R group potentials, resulting in a family of nine distinct, but related structures detected. The dkxanthenes are essential for viable-spore formation, and are also the major yellow pigment present in myxobacteria (Meiser et al., 2006). In addition, cittilin A (m/z of 631.275) and myxovirescin (m/z of 624.448), both non-ribosomal peptide secondary metabolites, were detected. Cittilin A is a peptide antibiotic with a structure derived from three tyrosines and an isoleucine residue, with no known cellular target (Grabley and Thiericke, 1999). Myxovirescin A (also called TA) has a macrocyclic structure and is an antibiotic that inhibits type II signal peptidases during protein secretion (Xiao et al., 2012).

**Table 3:** **Secondary metabolites identified in OMV fractions**.

**Name**	**Putative function**	**RT**	**m/z**	**Mass**	**Molecular formula**	**Compound ID**
Cittilin A	Antibiotic	4.5	631.275	630.268	C34H38N4O8	N/A
Dkxanthene 492 - 574	Yellow pigment	6.1–8.1	493.248–597.168^*^	492.238–574.280	C27H32N4O5-C32H38N4O6	N/A
Myxalamid A	Antibiotic	12.6	416.317	415.305	C26H41NO3	6440913
Myxalamid B	Antibiotic	11.7	402.301	401.291	C25H39NO3	5282085
Myxalamid C	Antibiotic	11.1	388.285	387.275	C24H37NO3	6439800
Myxochelin A	Chelating agent	4.7	405.167	404.16	C20H24N2O7	16093504
Myxochelin B	Chelating agent	2.7	404.183	403.177	C20H25N3O6	10873133
Myxovirescin A	Antibiotic	11.9	624.448	623.438	C35H61NO8	6450484

Metabolites were identified by LC MS analysis of OMV cell fractions, putative structures are shown in Figure 3 and detailed tandem MS data in Supplement Data S2. m/z values indicate [M+H] except when noted;

* denotes [M+Na].

### mepA is a metalloprotease with extracellular activity

**Figure 5:**
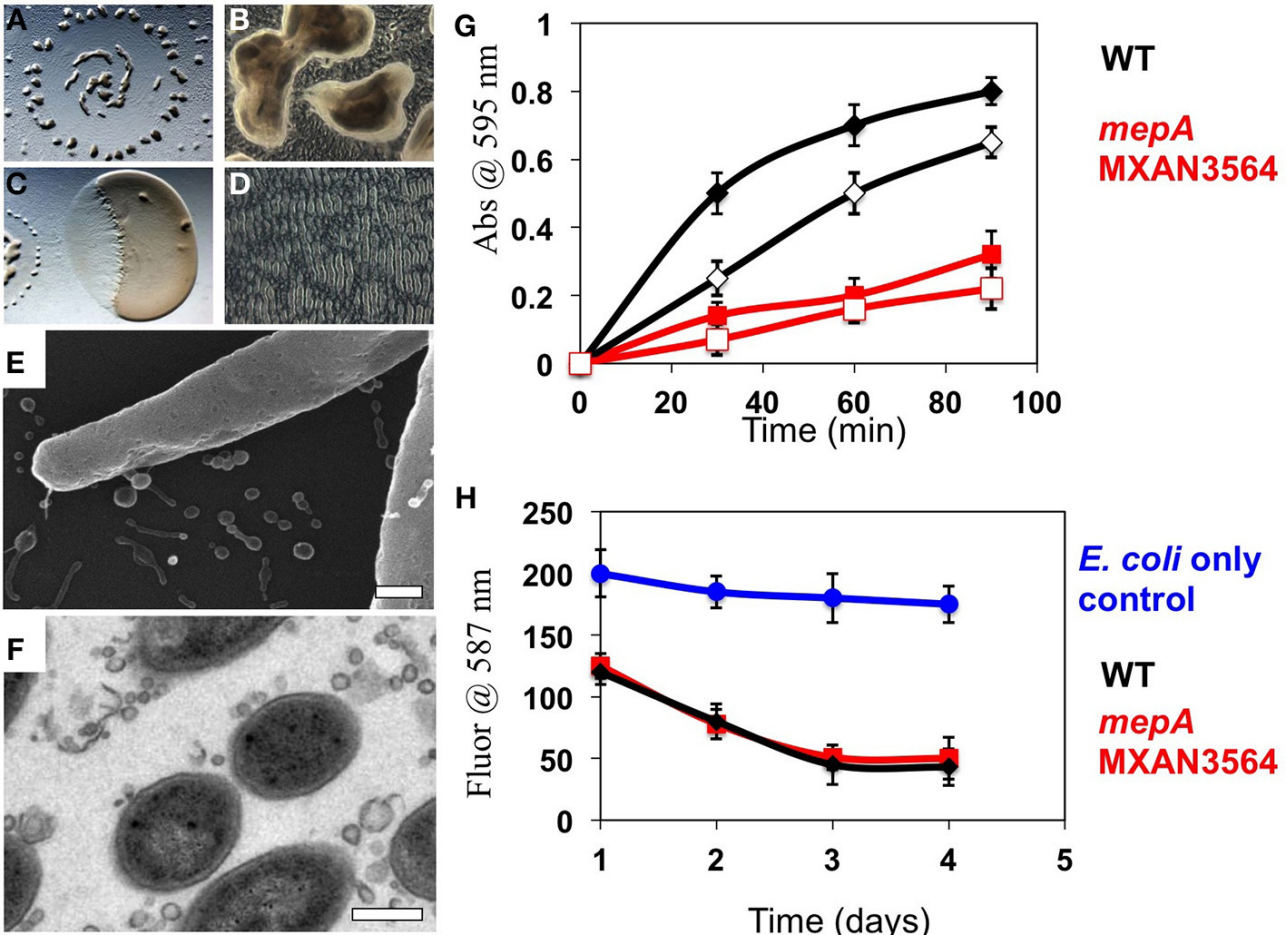
**Physiological analysis of a mepA metalloprotease mutant. (A,B)** 72 h incubation of a 10 μl aliquot of *mepA* cells induced to form fruiting bodies on CFL media. Normal fruiting body timing, distribution **(A)** and sporulation **(B)** were observed. **(C,D)** 48 h incubation of a 2 μl aliquot of *mepA* cells incubated adjacent to a 5 μl aliquot of *E. coli* on CFL media for observation of predatory behavior. Both clearing **(C)** and rippling **(D)** were observed. Electron microscopy of mepA cells shows the presence of vesicles with both **(E)** Scanning electron microscopy and **(F)** Transmission electron microscopy. **(G)** Resorufin-conjugated Casein was incubated with whole cell (solid marks) and vesicle preparations (open marks) of wild type DZ2 (black) and *mepA* (red) and proteolysis measured by the colorimetric release of soluble resorufin over 90 min, relative to control with no addition. *mepA* whole cells and OMV fractions show reduced proteolytic activity. **(H)** Analysis of prey cell lysis of mCherry expressing fluorescent *E. coli* cells by wild type DZ2 (black) and *mepA* (red) shows that both strains reduce fluorescent signal with similar kinetics, relative to the *E. coli* only control (blue). Error bars represent standard deviation from 3 independent assays, scale bars are 0.5 microns.

The presence of an M36 metalloprotease homolog (locus MXAN_3564), which we will subsequently refer to as *mepA*, at high abundance in OMV fractions led to the hypothesis that this protein could be the long-predicted, but to date elusive enzyme for enabling extracellular digestion of protein during wolf pack predatory behavior. This hypothesis prompted us to construct an *mepA* insertion mutant, disrupting MepA function. The *mepA* insertion mutant strain was subjected to thorough physiological analysis (see below). The *mepA* gene is located downstream of MXAN_3563, a putative nucleoside epimerase predicted to be involved in membrane biogenesis (note that MXAN_3563 was not detected here in either OMV or OM fractions) and upstream of MXAN_3565 a hypothetical protein coded for on the opposite DNA strand. The *mepA* mutant construct was cultured and analyzed for social behaviors by observing growth at high density on low nutrient CFL media. Fruiting bodies were observed to form with normal shape, distribution and timing in the *mepA* mutant, as expected (Figures 5A,B). When incubated in co-culture with *E. coli* prey on CFL media, the *mepA* mutant was observed to invade and lyse prey cells with normal timing and rippling behavior, indicating that it is not required for motility or lysis of prey (Figures 5C,D). We also examined *mepA* mutant cell cultures with SEM and TEM microscopy and observed the presence of OMV structures surrounding and attached to cells (Figures 5E,F).

To examine the putative proteolytic activity of the MepA protein, OMVs were isolated from both the *mepA* mutant and wild type DZ2. Using a resorufin labeled-casein protein substrate, we compared the protease activity of whole cells and purified OMVs in both strains (Figure 5G). Both whole cells and the OMV fractions of wild type showed rapid proteolytic activity, whereas the *mepA* mutant was deficient in both cases. The *mepA mutant* strain displayed only ~33 % of the rate of proteolysis compared to the wild type, suggesting that the *mepA* locus is an important part of the proposed extracellular protease cocktail produced by *M. xanthus*. It is unclear if this greatly reduced activity is due solely to MepA enzymatic activity or if loss of MepA has pleiotropic effects on other exoproteases of *M. xanthus*. It has previously been shown that OMVs mediate the killing of *E. coli* prey cells through the delivery of toxic proteins or antibiotics (Evans et al., 2012; Remis et al., 2014). Examination of these strains for lytic activity through incubation with mCherry-labeled *E. coli* cells indicated no difference between the rate of *E. coli* cell lysis by wild type *M. xanthus* and the *mepA* strain, suggesting that the protease activity of *mepA* is utilized only as a secondary factor during predation, by breaking down protein released by already lysed cells (Figure 5H).

## Discussion

In addition to the paradigm that Gram-negative cells have cellular compartments consisting of cytoplasm, inner membrane, periplasm, and outer membrane, our data suggests that *M. xanthus* cells possess another distinct compartment, i.e., outer membrane vesicles. We find that OMVs contain a unique protein cargo, distinguished from proteins found in the membranes, that predict extracellular hydrolytic functions for OMVs. This is in agreement with data showing functional activity associated with OMV fractions (Evans et al., 2012; Remis et al., 2014). The detection of OMV-enriched and OMV-specific proteins suggests the ability of *M. xanthus* to sort proteins into OMVs, but further study is required to determine if there is a protein sorting mechanism for OMVs of proteobacteria analogous to the COPII vesicle trafficking in eukaryotic cells (Zanetti et al., 2012). Bhat *et al*., have proposed that a lysine residue at position +7 in the N-terminal sequence directs proteins to the extracellular matrix (ECM), whereas a lysine residue at position +2 directs proteins to the IM (Bhat et al., 2011). No similarly conserved signal has yet been identified for OM proteins or OMV proteins, nor do we know the mechanism by which OMV structures are produced, but this data set should help focus future studies on OMV formation in *M. xanthus*. Previous studies indicate that *M. xanthus* OMV biogenesis occurs anywhere along the cell resulting either in pinching off single OMVs or long chains that stay attached to cells (Palsdottir et al., 2009; Remis et al., 2014). As our understanding of OMV content is refined we may be able to predict OMV-directed proteins in unstudied or understudied organisms.

OM and OMV compartments are likely critical to cell-cell signaling processes in both intraspecies and interspecies interactions (Krug et al., 2008; Mashburn-Warren et al., 2008; Evans et al., 2012). Several features are particularly striking, such as the presence of the MepA metalloprotease that, when disrupted, results in reduced exoenzyme proteolytic activity. Since there is still some exoprotease activity (33% of wildtype levels) detected in OMVs isolated from the *mepA* mutant, we expect that some of the other hydrolytic enzymes identified are also involved in macromolecule degradation. Since predation by bacteria requires both extracellular lysis of prey and acquisition of the nutrients released, we suggest that the MepA protein is likely involved in acquiring nutrients from released extracellular protein, but not in prey cell lysis. In addition, the high abundance of two GroEL chaperonin homologs within OMVs is intriguing. The importance of the two GroEL homologs in social behaviors such as fruiting body formation (Li et al., 2010) and antibiotic production (Wang et al., 2014) suggests that both proteins may exert these functions through localization to OMV organelles, and suggest that they are involved in ensuring proper folding of exoproteins after export. In addition, proteins involved in motility were detected in both the membrane and the OMV proteomes and, while supporting the idea that OMVs may play a role in the complex motility systems of *M. xanthus*.

The proteomics study employed a shotgun workflow that is based on a stochastic selection of precursor ions for tandem MS (MS/MS) upon which peptide/protein identification is based (McDonald and Yates, 2002). While very powerful as a proteomics discovery tool, the shotgun analytical format suffers from under-sampling (Frahm et al., 2006), thus limiting the achievable level of technical reproducibility (Tabb et al., 2010). When analyzing high complexity samples, a failure to detect/identify a protein under LCMS shotgun conditions does not preclude its presence in the sample but rather it suggests that a species might be present at a relatively low level. With this caveat in mind, a number of proteins were repeatedly identified in the same sample type, i.e., 45 and 31% of all OMV and membrane proteins, respectively were matched by at least two distinct peptides across all samples of a given category. We have assessed consistency of our data by evaluating two analytical attributes of the protein identification evidence, i.e., relative abundance (mol %) and a number of distinct peptides for their inter-sample variability. To this end, we used 128 and 242 OMV and membrane proteins, respectively, that were detected in three biological replicates by at least two distinct peptides. As shown in graphs 2A and 2B in S1, the CVs of relative abundances and distinct peptides numbers were below 0.6 for more than 90% of OMV and 70–90% OM identifications, suggesting high similarity among the samples. Likewise, we evaluated consistency of analysis of proteins between the sample types in the same biological replicate, this time comparing ratios between relative abundances and distinct peptide numbers in membrane and OMV fractions isolated from the same culture. Since in this case the results were affected by the analysis of twice as many samples (6 vs. 3), a larger spread of values was observed. Nevertheless, error factors remain below a two-fold level for more than 70% and ~90% of shared proteins when evaluating differences in relative abundances and numbers of distinct peptides, respectively (graph 2C in S1). Of note, a higher level of reproducibility was observed in the analysis of vesicles vs. membrane proteome, possibly due to the lower complexity of the former sample. While the above analyses highlight the significant similarity among the analyzed samples, targeted MS assays (Jaffe et al., 2008; Addona et al., 2009; Schmidt et al., 2009; Boja and Rodriguez, 2012) are required to validate the data presented here with a larger number of samples.

A diversity of secondary metabolites was also detected in the OMVs, many of which are thought to have antibiotic properties. These may assist in killing prey bacteria and also inhibiting the growth of completing microbes. Further study on the localization of these molecules is needed, but unlike classic antibiotic producers such as *Penicillium notatum*, *M. xanthus* typically kills upon cell-cell contact (Berleman and Kirby, 2009). The detection of several molecules with antibiotic properties leads to the hypothesis that targeted delivery of antibiotics through OMVs mediates prey cell lysis on contact. Such a mechanism would have the benefit that expensive secondary metabolites are not lost through diffusion. Furthermore, the packaging of several antibiotics and hydrolytic enzymes in a nano-scale package may provide a lethal cocktail that prevents the selection for single mutation resistance in the bacterial species that *M. xanthus* feeds on. If this holds true, then OMVs may be a powerful tool in the fight against multi-drug resistant pathogenic bacteria. Perhaps OMVs can be engineered to form a new generation of antibiotics—where susceptible cells are overwhelmed by a targeted cargo of multiple antibacterial molecules and hydrolytic enzymes. Further study of the protein and small molecule content on OMVs should improve our understanding of these organelles and their capacity to act as vehicles for intercellular delivery.

## Methods

### Cell growth and mutants

*M. xanthus* DZ2 (wild type) was grown in liquid culture shaking at 200 rpm, 32°C in CYE media consisting of 5 mM MOPS pH 7.6, 2 mM MgSO4, 0.5% (w/w) Bacto Casitone, 0.25% (w/w) Bacto Yeast Extract or on 1.5% agar plates with the same components.

### OMV and membrane purification

To obtain OMV and membrane cell fractions, 25 mL liquid *M. xanthus* cultures were first vortexed for 30 s to shear OMV structures from cells as before (Remis et al., 2014). The vortexed culture was then centrifuged for 10 min at 5000 × G to pellet the cells prior to decanting the OMV containing supernatant. The supernatant was then passed sequentially through a 0.45 μm filter followed by a 0.22 μm syringe filter to remove cellular debris that did not pellet with previous step. Finally the cell free liquid was centrifuged at 140,000 × G for 1 h and the resulting pellet resuspended in 1 mL of PBS to obtain OMV-fractions. For membrane fractions, the cell pellets were resuspended in PBS with 5 mg DNase I according to previous methods (Thein et al., 2010). The suspension was lysed by sonication, and centrifuged at 5000 × G to remove incompletely lysed cells. The suspension was centrifuged at 140,000 × G for 1 h and washed 3 times with PBS. Sample aliquots were subjected to electron microscopy analysis to monitor the presence and purity as well as possible contamination levels. All samples were resuspended and vesicles were purified as described above five independent times. Samples were mixed with lipid dye FM 4–64 (Molecular Probes) and using a microplate (Greiner Bio-One North America, Inc., Monroe, NC) and plate-reader (Tecan, Vission-100, AFAB Lab, LLC, Frederick, MD) relative concentration of vesicle sample was determined.

### Tryptic digestion and protein identification by LCMS

OMV and OM samples were digested using a method adopted from Papac et al. (1998), modified as previously described (Chhabra et al., 2011). A 5 μL aliquot of sample was then loaded onto a trapping cartridge (Dionex) and the sample washed for 10 min with 2% acetonitrile in 0.1% formic acid (10 μl/min). The trapping cartridge was then moved “in-line” with a C18 column analytical column (Dionex PepMap 75 μm i.d. × 150 mm) and peptide separation carried out using a 45-min linear gradient from 2 to 40% B at a flow rate of 600 nL/min (A: 2% acetonitrile (HPLC grade, Burdick and Jackson)/0.1% formic acid (Pierce Scientific); B: 98% acetonitrile/0.1% formic acid). The nano-LC-ESI-MSMS analysis utilized a 2D ultra NanoLC system (Eksigent) interfaced with a Thermo Scientific LTQ Orbitrap Velos (Waltham, MA) via an Advance CaptiveSpray Ionization source (Michrom BioResources, Auburn, CA) fitted with a Michrom BioResources CaptiveSpray 20 μm i.d. tapered tip. Nanospray was performed at a spray voltage of 1.5 kV. Precursor scans were acquired in the Orbitrap over the mass range of *m/z* 300–1500 with at a resolution of 30,000, with lock mass enabled. Twleve data-dependent MS/MS scans were performed per precursor scan using the LTQ. Charge state screening and monoisotopic precursor selection were enabled, and peptides with 1+ charge state and unassigned charge states were rejected. MS/MS were collected utilizing an isolation width of 3 Da, normalized collision energy of 30 eV, an activation Q of 0.250 and an activation time of 10 ms. The mass spectrometer was routinely calibrated with a solution of caffeine, MRFA peptide and Ultramark 1621 according to the manufacturer's instructions. MS/MS data were converted to a mascot generic format (.mgf) by Mascot Daemon software and Mascot v 2.2 search engine (Matrix Science) was employed to search the data against a custom *M. xanthus* protein sequence database (downloaded from UniProt) with common contaminants appended; a reversed version was concatenated to the database to follow false discovery rate (14862 sequences; 5626671 residues). Peaklists were generated with the Mascot Daemon (Matrix Sciences) using the “ThermoFinigan LCQ/DECA RAW file” (Thermo Fisher) data import filter; search was limited to doubly- and triply-charged precursors. The following search parameters were utilized: precursor mass tolerance 5 ppm; fragment mass tolerance 0.8 Da; tryptic digestion; 1 missed cleavage; fixed modification: Cys-carbamidomethyl; variable modifications: deamidation (Asn and Gln), Met-sulfoxide and Pyro-glu (N-term Gln). Average FDR values were 2.0 and 0.8% for OM and OMV samples, respectively. Proteins that were identified with at least one peptide scoring above the Mascot calculated identity threshold in at least one of the six analyzed samples are reported; no manual verification of MS/MS data was performed. Relative protein abundances were approximated by the protein molar fractions (mol %) that are based on the exponentially modified protein abundance index (emPAI) values that were automatically calculated by the Mascot search engine as described at the vendor's website <http://www.matrixscience.com/help/quant_empai_help.html> (Ishihama et al., 2005).

### Mass spectrometry of small molecules

OMV fractions were frozen and lyophilized overnight. The remaining powder was then collected in separate glass scintillation vials and 5 mL of a solvent mixture of 3:3:2 IPA:MeOH:H_2_O was added. The samples were then vortexed, and the liquid decanted to fresh Eppendorf tubes and dried down via speed vacuum. The extract was then reconstituted to 1 mL of the same solvent mixture. Precipitates were pelleted and liquid decanted to a new Eppendorf tube. These were once again speed vacuumed to dryness, reconstituted in 400 μl in the same solvent mixture, and filtered with 0.2 um filters. These samples were run on an Agilent 6550 mass spectrometer coupled with an Agilent UPLC Eclipse C18 column. A 21 min method was used with a C−18 column. Solvent A was LC MS grade water with 0.1% formic acid, and Solvent B was LC MS grade acetonitrile with 0.1% formic acid. Vesicle extract was diluted by 50% with solvent mixture, and 1 μl was injected. At a flow rate of 0.6 mL/min, the gradient used was 1 min at 5% Solvent B, and then a 19 min gradient to 95% Solvent B, followed by 1 min holding at 95% Solvent B. Metabolite profiling was performed via targeted searches for known *M. xanthus* secondary metabolites using Mass Hunter software (Agilent Technologies, Santa Rosa, CA). Ions matching known *M, xanthus* metabolites within 5 ppm were subjected to tandem MS to further support their identification by matching two or more known fragments.

### Mutant construction

Gene specific primers (Forward: 5′-CAGGCTTCACC-gene specific region-3′ and Reverse: 3′-AAAGCTGGGTC- gene specific region-5′) were designed to amplify ~500–600 bp of genomic coding region from genomic DNA. A second PCR was performed using Gateway® compatible primers (Forward: 5′-GGGGACAAGTTTGTACAAAAAAGCAGGCTTCACC-3′ and Reverse: 3′-GGGGACCACTTTGTACAAGAAAGCTGGGTC-5′). PCR products were run on a 1.5% agarose gel and excised under UV lamp. The gel, containing amplified genomic region were purified using QIAquick PCR purification kit (Qiagen). Purified DNA fragments were cloned into pDONR™201 using BP Clonase® II Enzyme Mix according to manufacturer's instructions (Life Technologies, USA) and incubated overnight at room temperature before transforming into chemical competent cells. DNA sequencing of the inserted gene was performed using flanking primers by Quintara Biosciences, USA.

### Electron microscopy

*Negative-staining by TEM. M. xanthus* cultures were fixed in 10% glutaraldehyde for 30 min and resuspended gently. 3 μL of this cell suspension was added to a formavar coated copper mesh grid (Electron Microscopy Sciences, Hatfield, PA) and then stained with 2 % uranyl acetate (3 μL) for 2 min. Excess cell suspension was adsorbed onto filter paper and washed with 3.5 μL ddH_2_O between additions. Whole mount negative stained samples were imaged at 60 kV on a JEOL 1200-EX transmission electron microscope.

### Scanning electron microscopy

Cultures were grown on poly-lysine coated silicon wafers in 2 ml CYE submerged cultures for 18 h at 32°C. Wafers were harvested and fixed with 2.5% Glutaraldehyde in Sodium Cacodylate buffer pH 7.2. Post-fixation was done on ice using 1% Osmium Tetroxide in Sodium Cacodylate buffer pH 7.2 for 1 h. Samples were dehydrated using a graded ethanol series (20, 40, 60, 80, 90, 100% × 3) at 10 min per step. Critical point drying was performed with a Tousimis AutoSamdri 815 Critical Point Dryer (Tousimis, Rockville, MD 20851, USA). Samples were then sputter coated with gold-palladium using a Tousimis Sputter Coater (Tousimis, Rockville, MD 20851, USA) to prevent charging in the microscope. Images were collected using the Hitachi S5000 Scanning Electron Microscope (Hitachi High Technologies America Inc, Pleasanton, CA 94588, USA).

*Thin section TEM. M. xanthus* colonies were grown for 4 days on CYE agar plates in 40 mm diameter aluminum weighing pans and prepared as before (Remis et al., 2014). Briefly, samples were fixed with 2.5% Glutaraldehyde for 1 h, post-fixed with 1% Osmium Tetroxide for and stained with 5 mM Ruthenium Red. Dehydration was performed using a graded ethanol series, followed by a step-wise infiltration with epon resin and polymerized in an 80°C oven. Sample blocks were sectioned at 90 nm using a Leica EM UC6 microtome (Leica Microsystems Inc. Buffalo Grove, IL). Sections were collected on slot grids and stained with 2% uranyl acetate and Reynolds lead citrate. Imaging was performed with an FEI Tecnai 12 transmission electron microscope (FEI, Hillsboro, OR).

### Physiological studies

Rapid fruiting body induction of *M. xanthus* and predation of *E. coli* strain β2155 were performed as before Berleman and Kirby (2007) by adding washed cells to CFL media and incubating at 32 C for 3–5 d. CFL contains 10 mM MOPS, pH 7.6, 1 mM KH_2_PO_4_, 8 mM MgSO_4_, 0.02% (NH_4_)_2_SO_4_, 0.002% Na Citrate, 0.01% Casitone and 0.002% Na Pyruvate. Images were captured using a Nikon SMZ1000 dissecting microscope (Nikon Instruments Inc., Melville, NY). Assays were performed at least three times, with representative images selected. For quantitative assays, *E. coli* strain β2155 harboring a constitutively expressed mCherry protein was utilized (Remis et al., 2014). Black Corning 96 well Polystyrol plate (Corning Life Sciences, Tewksbury, MA), with 200 μl of CFL agar were used for incubation and fluorescence measurement. 10 μl aliquots of *E. coli* cells were added and allowed to dry before adding 10 μl aliquots of *M. xanthus* DZ2 or *mepA* pipetted directly on the *E. coli*. Fluorescent measurements were aquired 1, 2, 3, and 4 days after plating with a Tecan Infinite M1000 microplate reader (Tecan Systems, Inc., San Jose, CA). Averaged readings across multiple samples were used to determine lytic rate.

Proteolysis of protein was determined using colorimetric detection of at abs = 574 nm of released resorufin-labeled casein protein (Roche, Inc., Mannheim, Germany). *M. xanthus* cultures or purified vesicles were incubated in 50 mM Tris-HCL pH7.8, 5 mM CaCl_2_ for 60 min at 37°C. To measure absorbance, aliquots were removed at the times indicated and proteolysis stopped through addition of 5 % Trichloroacetic acid. Remaining protein was pelleted through centrifugation and the supernatant assayed for released peptides. After 60 min incubation, An abs value of 0.2 is equivalent to 100 ng of trypsin hydrolysis.

### Conflict of interest statement

The authors declare that the research was conducted in the absence of any commercial or financial relationships that could be construed as a potential conflict of interest.
