# Supplementary material for: The lethal cargo of Myxococcus xanthus outer membrane vesicles
Source: Front Microbiol. 2014 Sep 9;5:474. doi: 10.3389/fmicb.2014.00474 (PMC4158809; doi:10.3389/fmicb.2014.00474)
Supplement: Supplementary file 2 [file DataSheet2.DOCX]

**Supplemental data**

**Supplemental data S1. Raw data from protein MS analysis.** (A-H) Eight spreadsheets are provided in a single workbook that details (A) Statistical analysis of the proteomics data obtained from 41,670 total peptide sequences (B) Evidence for each discrete protein ID, (C) 46 OMV-specific proteins metrics, (D) 188 OMV-enriched (shared with membrane fraction) protein metrics, (E) 314 Membrane fraction protein metrics, (F) Consistency analysis of OMV fraction, (G) Consistency analysis of Membrane fraction, (H) Consistency analysis between fractions..

**Supplemental Table S2.** Complete tandem MS data is provided, including Retention time (RT), m/z values, the parent mass and the fragmentation peaks observed for each identifiable secondary metabolite detected in OMV fractions.

| **Name** | **RT** | **m/z** | **Mass** | **Tandem MS peaks** |
| --- | --- | --- | --- | --- |
| Cittilin A | 4.5 | 631.275 | 630.268 | 603.278, 575.279, 518.181, 518.181, 490.192, 462.193, 445.165, 417.172, 292.154, 175.076 |
| Dkxanthene 492 | 6.1 | 493.248 | 492.238 | 374.21, 357.179, 390.162, 242.153, 225.126, 197.131, 390.200 |
| Dkxanthene 504 | 6.5 | 505.244 | 504.237 | 488.217, 427.197, 386.206, 369.179, 352.154, 254.153, 237.126, 209.131, 175.0843, 143.085 |
| Dkxanthene 508 | 5.9 | 509.238 | 508.231 | 492.211, 474.201, 418.207, 390.200, 373.174, 242.153, 225.127, 197.132, 175.084 |
| Dkxanthene 518 | 7.1 | 519.263 | 518.256 | 502.231, 400.220, 383.194, 366.168, 268.168, 251.142, 194.044, 175.086, 157.100, 143.084 |
| Dkxanthene 520 | 6.3 | 521.241 | 520.235 | 504.213, 486.202, 432.227, 402.202, 385.175, 254.154, 237.127, 209.1315, 175.085, 143.085 |
| Dkxanthene 544 | 8.1 | 545.276 | 544.270 | 528.249, 467.229, 426.237, 409.211, 332.161, 294.185, 277.158, 249.163, 221.130, 175.086 |
| Dkxanthene 548 | 7.2 | 549.269 | 548.262 | 532.242, 430.232, 413.204, 387.205, 368.184, 268.168, 251.142, 175.086, 143.084 |
| DKxanthene 560 | 6, 7.9 | 561.276 | 560.268 | 544.244, 526.233, 442.233, 425.207, 348.150, 294.185, 277.158, 248, 249.163, 227.066, 175.086 |
| Dkxanthene 574 | 8.0 | 597.268 | 574.280 | 558.258, 497.236, 456.247, 439.222, 413.223, 360.192, 318.182, 294.184, 277.158, 241.082, 175.086 |
| Myxalamid A | 12.6 | 416.317 | 415.305 | 398.305, 323.236, 290.211, 267.174, 208.133, 197.133, 187.148, 159.117, 145.101, 109.101 |
| Myxalamid B | 11.7 | 402.301 | 401.291 | 385.077, 361.315, 318.792, 283.260, 185.126, 159.115 |
| Myxalamid C | 11.1 | 388.285 | 387.275 | 370.273, 352.263, 313.214, 295.205, 267.209, 2215.143, 208.132, 187.147, 173.095, 159.116, 145.100, 133.100, 107.085 |
| Myxochelin A | 4.7 | 405.167 | 404.160 | 387.182, 307.112, 251.123, 216.133, 175.085 |
| Myxochelin B | 2.7 | 404.183 | 403.177 | 283.072, 245.103, 211.142, 387.150, 251.134 |
| Myxovirescin A | 11.9 | 624.448 | 623.438 | 592.420, 574.410, 556.399, 538.388, 520.377, 439.321, 421.310, 270.170, 424.282, 299.253, 366.337, 155.012 |
